# Analysis of epigenetic stability and conversions in *Saccharomyces cerevisiae* reveals a novel role of CAF-I in position-effect variegation

**DOI:** 10.1093/nar/gkt623

**Published:** 2013-07-17

**Authors:** Daniel C. B. Jeffery, Brandon A. Wyse, Muhammad Attiq Rehman, Geoffrey W. Brown, Zhiying You, Roxanne Oshidari, Hisao Masai, Krassimir Y. Yankulov

**Affiliations:** ^1^Department of Molecular and Cellular Biology, University of Guelph, Guelph, Ontario, Canada, ^2^Laboratory for Foodborne Zoonoses, Public Health Agency of Canada, Guelph, Ontario, Canada and ^3^Department of Genome Medicine, Genome Dynamics Project, Tokyo Metropolitan Institute of Medical Science, Tokyo, Japan

## Abstract

Position-effect variegation (PEV) phenotypes are characterized by the robust multigenerational repression of a gene located at a certain locus (often called gene silencing) and occasional conversions to fully active state. Consequently, the active state then persists with occasional conversions to the repressed state. These effects are mediated by the establishment and maintenance of heterochromatin or euchromatin structures, respectively. In this study, we have addressed an important but often neglected aspect of PEV: the frequency of conversions at such loci. We have developed a model and have projected various PEV scenarios based on various rates of conversions. We have also enhanced two existing assays for gene silencing in *Saccharomyces cerevisiae* to measure the rate of switches from repressed to active state and *vice versa*. We tested the validity of our methodology in *Δsir1* cells and in several mutants with defects in gene silencing. The assays have revealed that the histone chaperone Chromatin Assembly Factor I is involved in the control of epigenetic conversions. Together, our model and assays provide a comprehensive methodology for further investigation of epigenetic stability and position effects.

## INTRODUCTION

Gene silencing is mediated by compact heterochromatin, which is re-established after each passage of the replication fork (1–3). At certain loci, rare conversions between the silenced and active states of genes confer a quasi-stable pattern of gene expression called position-effect variegation (PEV) (4,5). PEV and the underlying epigenetic conversions can play a significant role in the adaptation of many single cell eukaryotes (including pathogens like *Trypanosome* and *Plasmodium*) to the changing environment (6). In metazoans, epigenetic stability is critical for cell differentiation and development, whereas untimely epigenetic conversions have been linked to cancer and to various genetic and psychiatric disorders (7–11).

The mechanisms of conversion that produce PEV are not well understood. In model organisms (*Drosophila melanogaster*, *Saccharomyces cerevisiae* and *Schizosaccharomyces pombe*), the expression of otherwise silenced genes can be increased by mutations in many other genes. However, in many cases, it is not clear whether these effects are caused by poor maintenance and leaky expression of the gene of interest or by elevated rates of epigenetic conversions or both (3,12). On the other hand, many suppressors of PEV expand or contract the heterochromatin domain, thus repositioning the variegated locus (6,13) with no clear evidence that the switching mechanism is affected. The main reason for this feeble grasp on the nature of epigenetic conversions is the rare use of direct quantitative assays.

Most tests assess variegation at the endpoint of a multi-generational process, but not the dynamics of conversions itself. These tests include the calculation of the proportion of *URA3-*expressing cells in *S. cerevisiae* or *URA4* in *S. pombe* after a certain period of growth in non-selective medium; the scoring of pink segmentation of yeast colonies caused by the variegated repression of *ADE2* in *S. cerevisiae* (*ADE4* in *S. pombe*) or the scoring of the patchy red coloring of the *D. melanogaster* eye caused by positional silencing of the *white* gene (14). In all these cases, the proportion of cells with active versus silenced genes has been recorded, but the process that led to the variegated state has not been directly studied. Only a few reports have closely looked at the dynamics of epigenetic switches. An early article has extensively analyzed the maintenance and ‘decay’ of gene silencing at the mating *HMLα* locus of *S. cerevisiae* (15). The authors have used sophisticated mating assays such as ‘shmoo’ farming and alpha-factor confrontation to determine the rates of conversion of epigenetic states and to provide a framework for future analyses (15). More recent studies of the mating type loci have used green fluorescent protein (GFP)-reporters to focus on the early events in the establishment and decay of gene silencing at a single cell resolution (16–18). Interestingly, the rates of epigenetic conversions measured by these short-term and long-term assays are considerably different. Finally, in certain histone mutants, an enhanced stability of epigenetic state at the telomeres of *S. cerevisiae* has been observed (19,20), but the authors have not elaborated on the rates of conversion.

In *Plasmodium falciparum*, a sophisticated system of silencing and switching of 60 subtelomeric *var* genes contributes to allelic exclusion phenotypes and immune evasion. These processes are believed to be responsible for the persistence of malaria infections and have attracted significant attention in recent years (21,22). Several studies have assessed the on- and off- rates of *var* genes through a complex algorithm and the measurements of the abundance of different *var* RNAs or different surface antigens in cloned cultures (23–26).

Here, we have generated a simple model for the quantification of conversions at any PEV locus and used this model to predict patterns of variegated expression based on the frequency of active→silenced (A→S) and silenced→active (S→A) transitions of a gene. We have expanded and substantially modified two existing assays for the assessment of gene silencing at the telomeres (27) or at the mating type loci (28) of *S. cerevisiae* and have fit the data to theoretical curves based on this model. These assays have given novel insights into epigenetic conversions and have confirmed the role of Chromatin Assembly Factor I (CAF-I) in these processes.

## MATERIALS AND METHODS

### Strains, growth conditions and integrating constructs

All strains used are listed in Table 1. As many of the used strains were temperature sensitive, all cells were grown at 23°C in non-selective (1% yeast extract, 2% tryptone, 2% glucose YPD) or on synthetic complete (SC) media lacking Uracil (SC-ura) or Arginine (SC-arg) or SC containing 0.1% or 0.2% 5-Fluoro-Orotic Acid (SC/FOA), 10 mM Hydroxyurea (HU) or 60 µg/ml canavanine as indicated. FOA was from Toronto Research Chemicals Inc. HU and canavanine were from Sigma-Aldrich. The experiments with *pol30* mutants were conducted in media without Tryptophan (SC-trp). These mutants lack the genomic copy of *POL30*, and their viability is maintained by plasmids (pRS, *ARS1, CEN4, TRP1*) carrying mutant or wild-type copies of *POL30* (36).

**Table 1.** Strains used in this study

Strain	Genotype	Source
*W303*	*MATa ade2-1 his3-11,15 leu2-3, 112 trp1-1 ura3-1 can1-100*	Open Biosystems
*Δcac1*	*W303 MATa cac1::LEU2*	(29)
*ZGY450*	*W303 MATα cac1::LEU2 hmr::GFP*	(30)
*ZGY491*	*W303 MATα cac1::LEU2, rtt106::kanMX, hmr::GFP*	(30)
*ZGY460*	*MATα cac1::LEU2, hir1::KanMX, hmr::GFP*	(30)
*ZGY459*	*MATa cac1::LEU2, asf1::KanMX6, hmr::GFP*	(30)
*Orc2-1*	*W303 orc2-1 MAT***a** *ts at 37°C*	(31)
*Orc5-1*	*W303 orc5-1 MAT***a** *ts at 37°C*	(32)
*Mcm5-1*	*W303 mcm5-1 MATα ts at 37°C*	(33)
*Mcm5-461*	*W303 mcm5-461 MATα ts at 37°C*	(33)
*Cdc45-1*	*W303 cdc45-1 MAT***a** *ts at 15°C*	(34)
*Cdc6-1*	*W303 cdc6-1 MAT***a** *ts at 37°C*	(35)
*pol30-0*	*W303 pol30Δ URA3-VIItel + (pBL230-POL30 TRP1) MATα*	(36)
*pol30-6*	*W303 pol30Δ URA3-VIItel + (pBL230-pol30-6 TRP1) MATα*	(36)
*pol30-8*	*W303 pol30Δ URA3-VIItel + (pBL230-pol30-8 TRP1) MATα*	(36)
*pol30-79*	*W303 pol30Δ URA3-VIItel + (pBL230-pol30-79 TRP1) MATα*	(36)
*Δsir1*	*W303 ade2-1, sir1Δ::LEU2 MATa*	(16)
*hmr-a1Δ::URA3*	*W303 ade2-1 hmr-a1Δ::K.l.URA3 MAT***a**	(16)
*Δsir1 hmr-a1Δ::URA3*	*W303 ade2-1 sir1Δ::LEU2 hmr-a1Δ::K.l.URA3 MAT***a**	(16)
*Δdot1 hmr-a1Δ::URA3*	*W303 ade2-1 dot1Δ::HIS3 hmr-a1Δ::K.l.URA3 MAT***a**	(16)
*BY4742*	*MATα leu2 ura3 his3 lys2*	Open Biosystems
*ΔΔcac1*	*BY4742 MATa cac1::kanMX*	Open Biosystems
*Δasf1*	*BY4742 asf1Δ::KanMX*	Open Biosystems
*Δhir1*	*BY4742 hir1Δ::KanMX*	Open Biosystems
*Δrtt106*	*BY4742 rtt106Δ::KanMX*	Open Biosystems
*Δasf1Δcac1*	*BY4742 asf1Δ::KanMX cac1Δ::LEU2 MATα*	(28)
*Δrtt106Δcac1*	*BY4742 rtt106Δ::KanMX cac1Δ::LEU2 MATα*	(28)
*Δhir1Δcac1*	*BY4742 hir1Δ::KanMX cac1Δ::LEU2 MATα*	(28)
*Δasf1Δhir1*	*BY4742 rtt106Δ::KanMX hir1Δ::LEU2 MATα*	(28)
*Δsas2*	*BY4742 sas2Δ::KanMX*	Open Biosystems
*Δsas3*	*BY4742 sas3Δ::KanMX MATα*	Open Biosystems
*Δrif1*	*BY4742 rif1Δ::KanMX MATα*	Open Biosystems

The integrating constructs *ADH4-URA3-tel*, *VR-URA3-tel* and *ADH4-ADE2-URA3-tel* were produced by digestion of pUCAIV or pUCAV with *Sal*I and *Eco*RI as described (27), and cells were transformed by electroporation. Correct telomeric integration was confirmed by PCR and by growth on both SC-ura and SC/FOA plates.

The number of generations of each culture was calculated as follows. Single colonies of freshly transformed cells were inoculated in 3 ml of YPD and grown to saturation. In this initial liquid culture, the cells undergo between 8 and 12 doublings. Subsequently, the liquid cultures were routinely diluted 1000 times and re-grown to saturation. The OD_600_ of each saturated culture was measured and used to calculate the exact number of generations in each passage of the culture: number of generations = [log(Final OD_600_/Initial OD_600_)]/log(2).

### Telomere position effect assays using *URA3*

Cells were transformed with the integrating constructs and selected for *URA3* expression by two consecutive passages on SC-ura plates. The cells were then transferred to non-selective YPD medium to allow for unrestricted A→S transitions and silencing of *URA3* and then spread on both SC-ura and SC/FOA plates. Three single colonies from each plate were inoculated in 3 ml of YPD media and grown for 10–40 generations. Aliquots from these cultures were serially diluted and spotted on YPD, SC-ura and SC/FOA plates. After 4 days of growth at 23°C, the colonies on the plates were counted and used to calculate the percentage of cells growing on SC-ura (these represent cells with active *URA3*) and SC/FOA (these represent cells with silenced *URA3*). The average values and standard deviations from all experiments are shown in Table 2.

**Table 2.** Measurements of the %FOA^R^ and %URA^+^ cells after selection on SC/FOA and SC-ura plates

	Initial selection on SC/FOA						Initial selection onSC-ura					
	%URA^+^	STD		%FOA^R^	STD		%URA^+^	STD		%FOA^R^	STD	
*VIIL telomere*												
*wt (BY4742)*	66%	11%	(*n* = 15)	39%	9%	(*n* = 15)	59%	7%	(*n* = 15)	45%	9%	(*n* = 15)
*Δrif1*	33%	3%	(*n* = 9)	68%	12%	(*n* = 9)	22%	14%	(*n* = 9)	70%	10%	(*n* = 9)
*Δsas2*	93%	11%	(*n* = 9)	3%	11%	(*n* = 9)	93%	10%	(*n* = 9)	4%	1%	(*n* = 9)
*Δsas3*	90%	17%	(*n* = 9)	1%	1%	(*n* = 9)	94%	17%	(*n* = 9)	3%	1%	(*n* = 9)
*wt (W303)*	60%	4%	(*n* = 15)	38%	4%	(*n* = 15)	65%	10%	(*n* = 15)	41%	10%	(*n* = 15)
*orc2-1*	83%	4%	(*n* = 12)	1%	1%	(*n* = 12)	83%	5%	(*n* = 12)	1%	0.04%	(*n* = 12)
*orc5-1*	89%	3%	(*n* = 12)	5%	2%	(*n* = 12)	93%	5%	(*n* = 12)	1%	1%	(*n* = 12)
*mcm5-461*	93%	3%	(*n* = 12)	11%	3%	(*n* = 12)	92%	3%	(*n* = 12)	12%	4%	(*n* = 12)
*bob1-1*	95%	4%	(*n* = 12)	5%	4%	(*n* = 12)	90%	5%	(*n* = 12)	3%	3%	(*n* = 12)
*cdc45-1*	91%	8%	(*n* = 12)	0.3%	0.2%	(*n* = 12)	91%	14%	(*n* = 12)	4%	5%	(*n* = 12)
*cdc6-1*	77%	11%	(*n* = 12)	6%	1%	(*n* = 12)	70%	3%	(*n* = 12)	2%	0.3%	(*n* = 12)
*Δcac1*	1%	1%	(*n* = 15)	93%	4%	(*n* = 15)	94%	3%	(*n* = 15)	1%	3%	(*n* = 15)
*Δasf1*	64%	6%	(*n* = 9)	29%	7%	(*n* = 9)	84%	8%	(*n* = 9)	20%	9%	(*n* = 9)
*Δhir1*	79%	7%	(*n* = 9)	58%	5%	(*n* = 9)	93%	4%	(*n* = 9)	53%	11%	(*n* = 9)
*Δrtt106*	13%	1%	(*n* = 9)	95%	5%	(*n* = 9)	91%	15%	(*n* = 9)	21%	4%	(*n* = 9)
*Δcac1Δasf1*	1%	1%	(*n* = 6)	96%	4%	(*n* = 6)	94%	6%	(*n* = 6)	2%	2%	(*n* = 6)
*Δcac1Δhir1*	1%	0.4%	(*n* = 6)	95%	9%	(*n* = 6)	103%	15%	(*n* = 6)	2%	2%	(*n* = 6)
*Δcac1Δrtt106*	8%	2%	(*n* = 6)	89%	11%	(*n* = 6)	92%	11%	(*n* = 6)	2%	2%	(*n* = 6)
*Δhir1Δasf1*	80%	5%	(*n* = 6)	52%	12%	(*n* = 6)	72%	9%	(*n* = 6)	50%	3%	(*n* = 6)
*pol30-0*	46%	9%	(*n* = 3)	39%	14%	(*n* = 3)	62%	13%	(*n* = 3)	35%	6%	(*n* = 3)
*pol30-6*	94%	14%	(*n* = 3)	8%	2%	(*n* = 3)	105%	18%	(*n* = 3)	8%	3%	(*n* = 3)
*pol30-8*	58%	12%	(*n* = 3)	16%	6%	(*n* = 3)	88%	18%	(*n* = 3)	2%	1%	(*n* = 3)
*pol30-79*	96%	7%	(*n* = 3)	1%	1%	(*n* = 3)	77%	8%	(*n* = 3)	1%	0.3%	(*n* = 3)
*Δsir1*	53%	5%	(*n* = 3)	54%	13%	(*n* = 3)	62%	16%	(*n* = 3)	55%	8%	(*n* = 3)
*VR telomere*												
*wt (BY4742)*	64%	15%	(*n* = 3)	22%	8%	(*n* = 3)	51%	9%	(*n* = 3)	28%	4%	(*n* = 3)
*Δcac1*	0.21%	0.04%	(*n* = 3)	109%	20%	(*n* = 3)	89%	12%	(*n* = 3)	0.06%	0.03%	(*n* = 3)
*hmr-a1Δ::URA3*												
*wt (W303)*	0.002%	0.002%	(*n* = 3)	91%	23%	(*n* = 3)	0.005%	0.001%	(*n* = 3)	80%	12%	(*n* = 3)
*Δsir1*	62%	16%	(*n* = 3)	53%	14%	(*n* = 3)	97%	10%	(*n* = 3)	1%	0.1%	(*n* = 3)
*Δdot1*	0.003%	0.002%	(*n* = 3)	78%	17%	(*n* = 3)	0.2%	0.3%	(*n* = 3)	86%	5%	(*n* = 3)

Details on the measurements and the calculations are provided in the text.

### Assay for gene silencing at *HMR*a using GFP

Strains containing a GFP expression cassette in the *HMR***a** locus (30) were grown in YPD and briefly sonicated to disperse cell aggregates. The culture was then diluted to 1 cell/ml, and 200 µl of aliquots were dispensed in several 96-well plates. The plates were incubated overnight at 23°C, until a single cluster of cells could be seen at the bottom of the well. Under these conditions, most of the mini-cultures originate from a single cell and go through ∼15 generations. Each well containing a single cluster of cells was analyzed by fluorescence-activated cell sorting (FACS) to determine the percentage GFP-fluorescing cells.

## RESULTS

### Model and rationale

**Figure 1.**
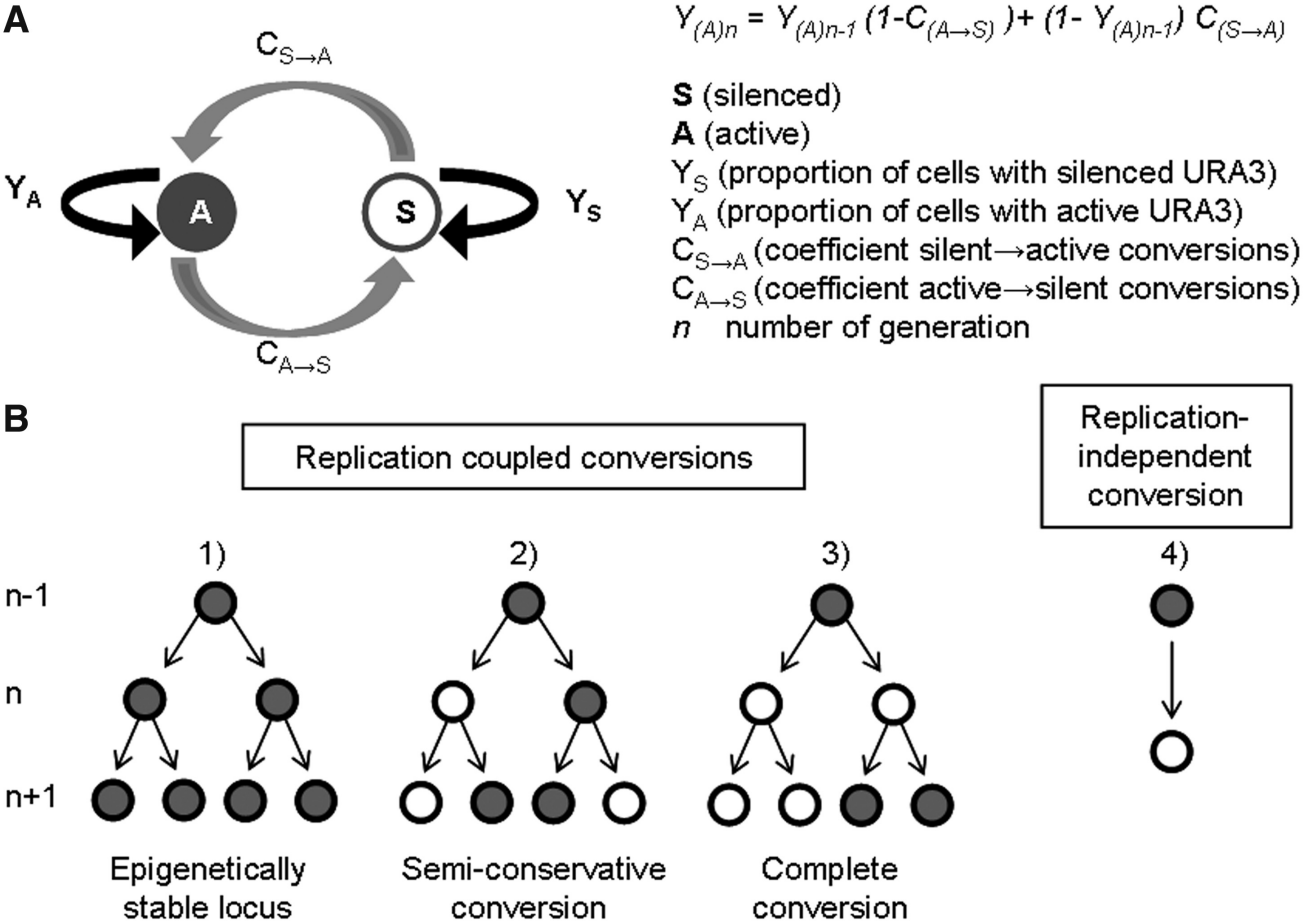
A general model for PEV. (**A**) A diagram showing the proportion of cells with the active and the silent gene at a PEV locus is shown. The conversion rates between silent and active state are depicted by gray bend arrows. The ‘conservative’ transmission of the two states is shown in black arrows. The formula for the calculation of the proportion of cells with active gene *(Y_A_)* in any given *n* generation is shown on the right. (**B**) A diagram of possible replication-coupled and replication-independent transmissions and conversions of a gene at a PEV locus is shown. Our model does not distinguish between these scenarios.

Positional variegation is defined by infrequent A→S and S→A conversions of a gene. We recaptured this process through modeling and simulation. A diagram depicting the parameters we used is shown in Figure 1. A distribution algorithm was applied to calculate the proportion of cells with silenced (*Y_S_*) and active (*Y_A_*) gene in a given generation (*n* − *1, n, n + 1 *…) based on the proportion of cells with silenced and active gene in the preceding generation and two coefficients of conversions (*C_S→A_* and *C_A→S_*) (Figure 1A). The equations and related calculations are presented in Appendix 1 (Supplementary Materials). We used the formula **Y_(A)n_ = Y_(A)n-1_−Y_(A)n-1_C_(A→S)_ + (1 −Y_(A)n__−__1_)C_(S→A)_** to simulate the frequency of conversions at any PEV locus. Y_(A)n_ is the proportion of cells with an active gene in any generation (*n* − *1, n, n + 1 …*), C_(A→S)_ is the coefficient of conversions from active to silent state and C_(S→A)_ is the coefficient of conversions from silent to active state. The analysis of this recurrence relation (Appendix 1) shows that the rates of conversion (*C_S→A_* and *C_A→S_*) must be between 0 and 100%, and that they are independent of the proportions of cells with silent (*Y_S_*) and active (*Y_A_*) gene. More importantly, they are independent of each other. This formula allows for the projection of experimental outcomes based on the *C_S→A_* and *C_A→S_* conversion rates and fixed initial values of Y_(A)0_. It also provides means for the calculation of the *C_S→A_* and *C_A→S_* rates based on experimental data. The calculated *C_S→A_* and *C_A→S_* values represent a direct measure of the ‘A→S’ and ‘S→A’ rates.

**Figure 2.**
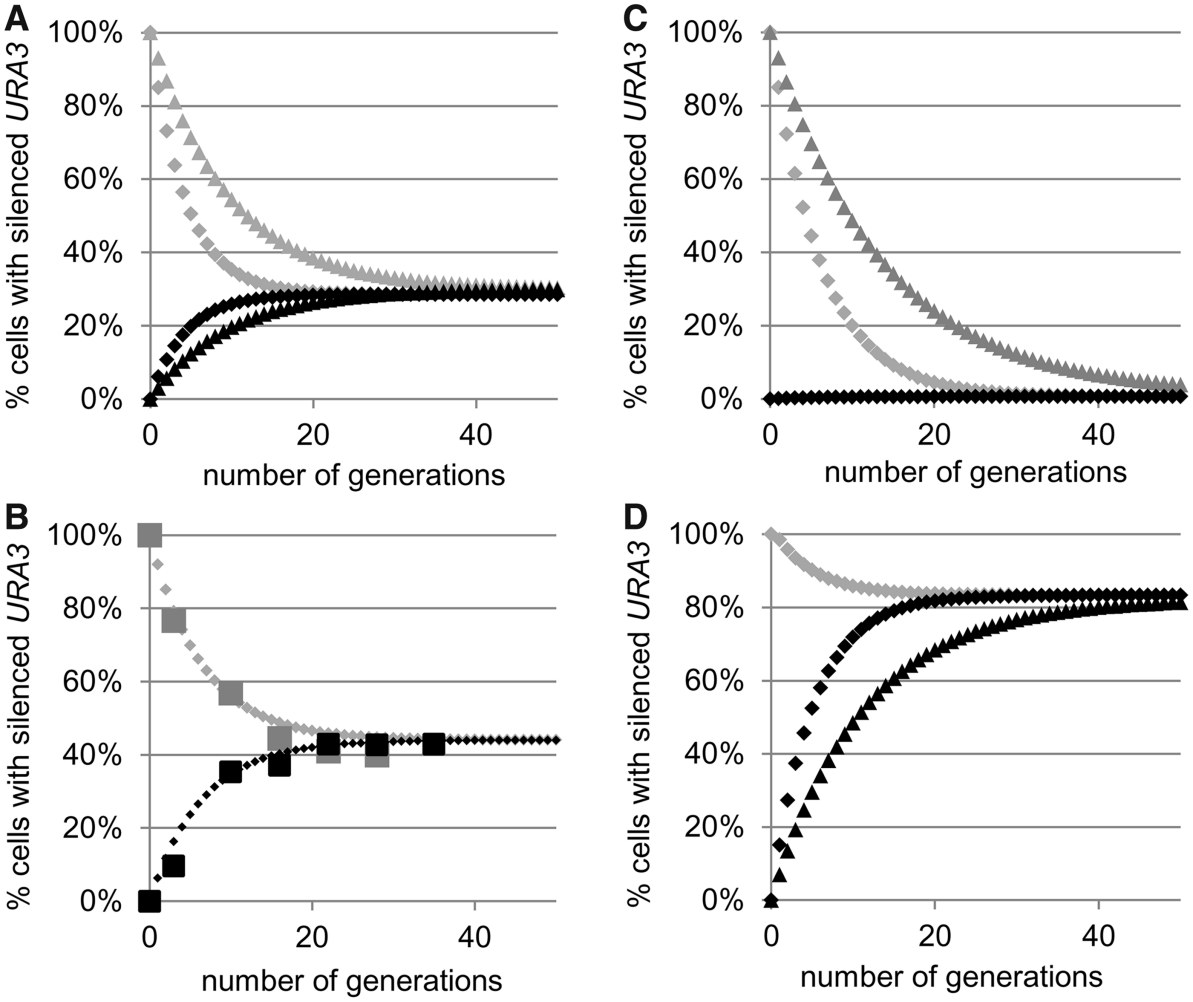
Simulation of PEV and calculation of *C_A→S_* and *C_S→A_*. (**A**) Simulation of the conversions of *URA3* after selection of SC/FOA plates (gray diamonds or triangles) or SC-ura (black diamonds or triangles) at *C_S→A_* = 15% and *C_A→S_* = 6% (gray and black diamonds) or *C_S→A_* = 7% and *C_A→S_* = 3% (gray and black triangles). (**B**) Best fit analysis of the conversion rates in wild-type (*W303*) cells. Cells were selected on SC/FOA (large gray squares) and SC-ura plates (large black squares) and transferred to YPD medium. Aliquots were taken out at known generation numbers, and the percentage FOA^R^ cells were measured and plotted. Best fit algorithm (small diamonds) produced values of *C_S→A_* = 8.0% and *C_A→S_* = 6.3%. (**C**) Simulation of loss of gene silencing. Simulations are similar to those in Figure 2A. The following values were used: *C_S→A_* = 15% and *C_A→S_* = 0.01% (gray and black diamonds) or *C_S→A_* = 7% and *C_A→S_* = 0.01% (gray triangles). (**D**) Simulation of gain of gene silencing. Simulations are similar to those in Figure 2A. The following values were used: *C_S→A_* = 3% and *C_A→S_* = 15% (gray and black diamonds) or *C_S→A_* = 3% and *C_A→S_* = 7% (black triangles).

**Figure 3.**
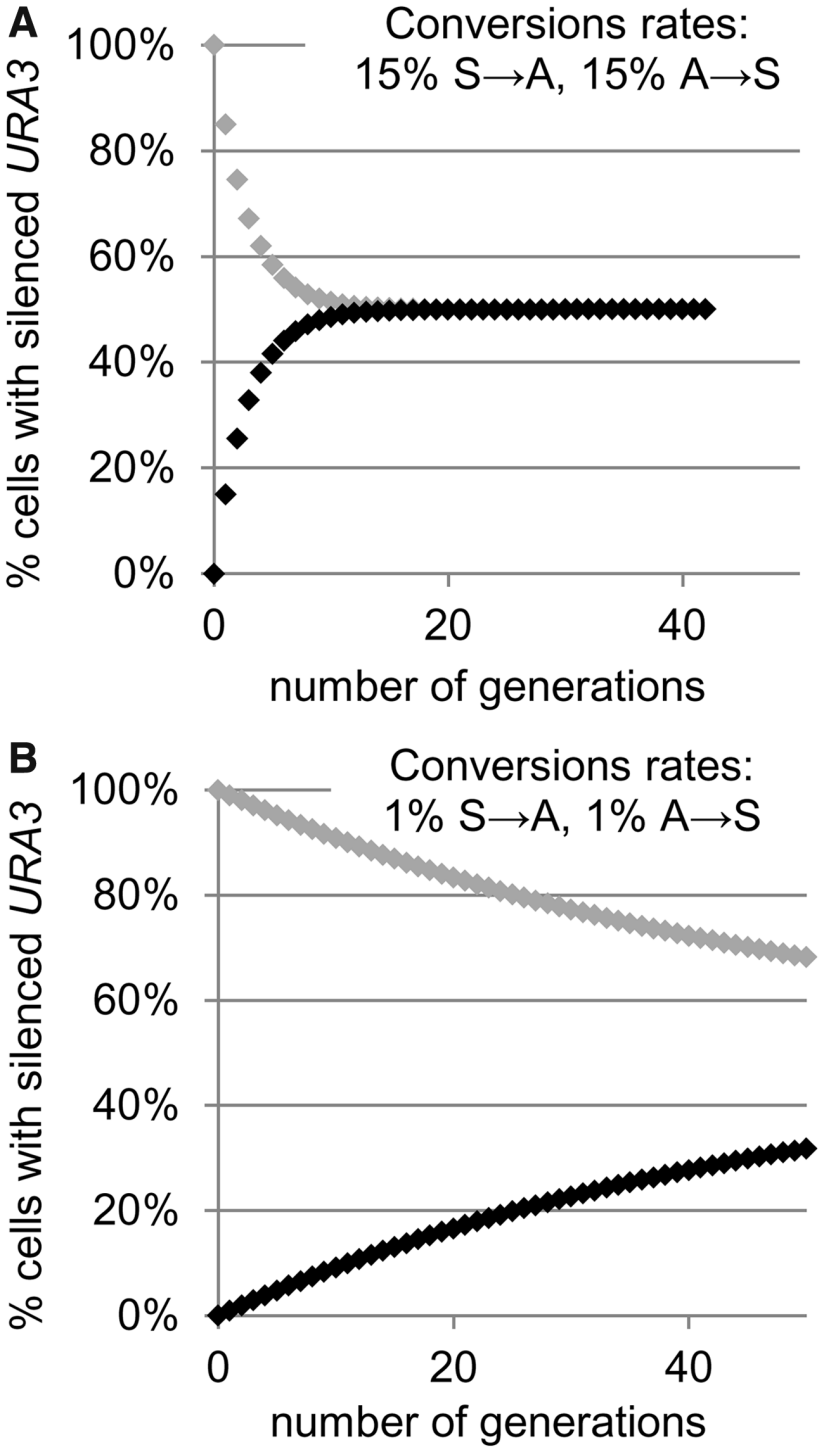
Simulation of gain and loss of epigenetic stability. (**A**) Loss of epigenetic stability. Simulations similar to Figure 2A with *C_S→A_* = 15% and *C_A→S_* = 15% (gray and black diamonds). (**B**) Gain of epigenetic stability. Simulations similar to Figure 2A with *C_S→A_* = 1% and *C_A→S_* = 1% (gray and black diamonds).

**Figure 4.**
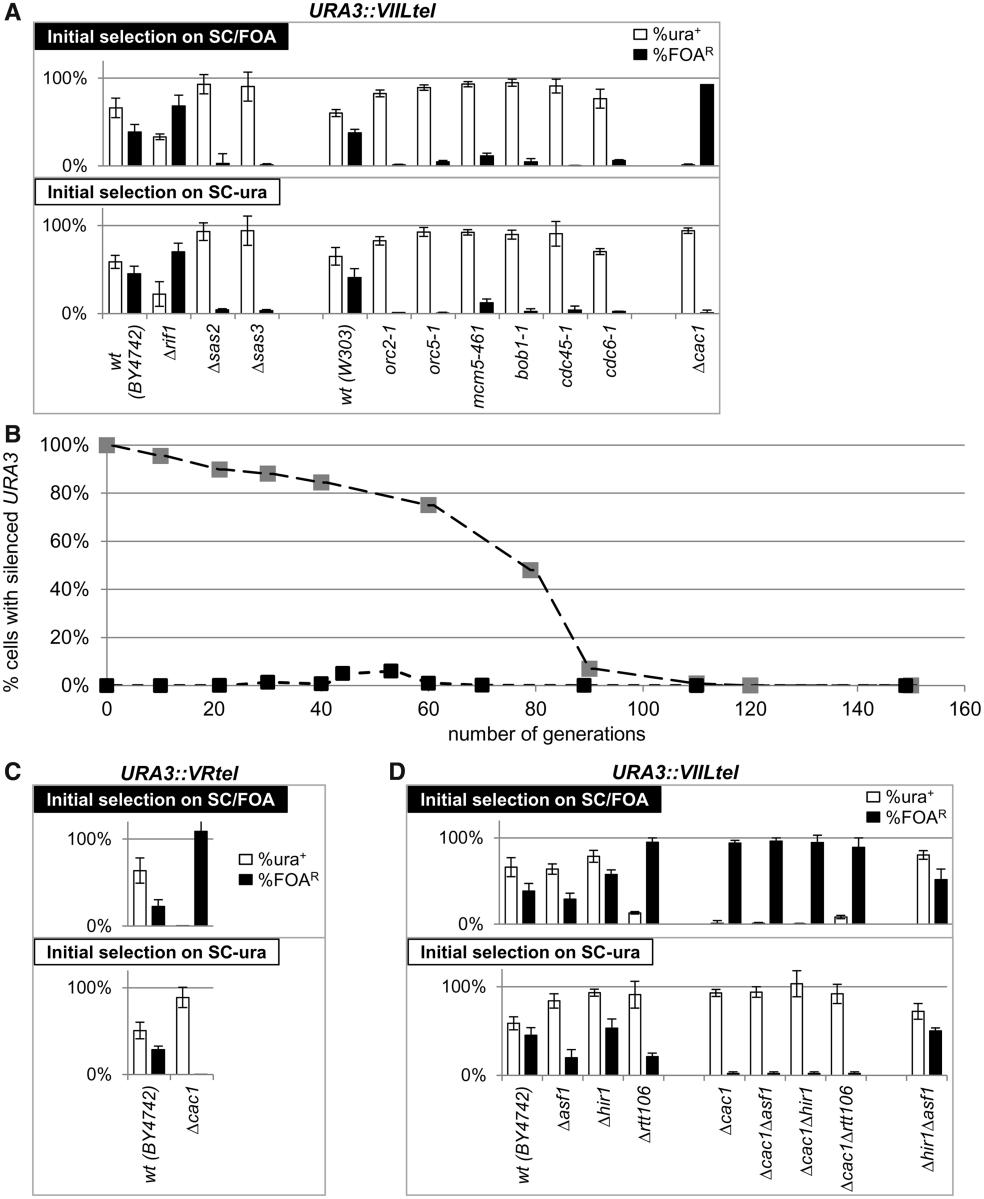
Assessment of the frequency of conversions at the *VIIL* and *VR* telomeres. (**A**) Frequency of conversions at the *VIIL* telomere. *URA3* was inserted in the *VIIL* telomeres of the strains shown below the graphs. Cells were selected on SC/FOA (upper graph) or SC-ura (lower graph) and single colonies were transferred to YPD medium and grown for ∼15–20 generations at 23°C. The cultures were serially (1:10) diluted and spotted on SC, SC-ura and SC/FOA plates. The colonies were counted, and the percentage of URA^+^ (open columns) and FOA^R^ (black columns) cells were calculated for at least six independent cultures and plotted. Data are from Table 2. (**B**) Long-term rates of conversion in *Δcac1* cells. Cells were selected as in Figure 4A and grown for 160 generations. Aliquots of the cultures were removed at known generations, and the percentage FOA^R^ cells was measured and plotted. Best fit algorithm of the conversion rates for the first 50 generations produced values of *C_S→A_* = 0.44% and *C_A→S_* = 0.06%. Conversion rates in later generations were not measured. One of two independent long-term experiments is shown. (**C**) Frequency of conversions at the *VR* telomere. *URA3* was inserted in the *VR* telomeres of *BY4742* and *Δcac1* strains. Analyses were performed as in Figure 4A. (**D**) Frequency of conversions at the *VIIL* telomere in single and double histone chaperone mutants. Analyses were performed as in Figure 4A.

This model does not address the precise nature of conversions as shown by the ‘branching’ diagrams in Figure 1B. For example, during DNA replication none, one or both of the duplicated genes can maintain the pre-existing state (‘A’ or ‘S’). Our model does not distinguish if one or both progeny genes convert in a single step (Figures 1B, 2 and 3). In addition, our model does not distinguish between replication-coupled (Figures 1B, 2 and 3) and replication-independent (Figures 1B and 4) conversions.

### Simulation of telomeric PEV

Most telomere position effect (TPE) assays in *S. cerevisiae* are conducted by the insertion of *URA3* next to the left telomere of chromosome *VII* (27). After selection on plates without uracil (SC-ura, this corresponds to Y_(A)0_ value close to 100%), the cells are grown in non-selective medium for 20 or more generations to allow for unrestricted A→S and S→A conversions. It is assumed, but rarely confirmed, that at this time point, the culture has reached a dynamic equilibrium of cells with silenced (*Y_S_*) and active (*Y_A_*) *URA3*. Subsequently, aliquots of these cultures are applied to plates containing 5-FOA (SC/FOA) and to non-selective SC plates. 5-FOA is a non-toxic precursor of uridine monophosphate (UMP) synthesis, but is turned into highly toxic 5-FUMP by the *URA3*-encoded Orotidine-5′-phosphate-decarboxylase. Any cell expressing *URA3* at the time of plating will die on SC/FOA, whereas cells with repressed *URA3* will produce a colony (14). Therefore, the level of *URA3* silencing is assessed by the number of colonies on SC/FOA divided by the number of colonies on non-selective plates [% FOA-resistant cells (FOA^R^)].

In wild-type cells, such TPE experiments produce equilibrium in the vicinity of 30% FOA^R^/70% FOA-sensitive (FOA^S^) cells (37–39). If the initial value of Y_(A)0_ is set at 100% (corresponding to selection on SC-ura plates) or at 0% (corresponding to selection on SC/FOA plates), this equilibrium will be reached in 20 generations with *C_S→A_* = 15% and *C_A→S_* = 6%. Values of *C_S→A_* = 7% and *C_A→S_* = 3% will produce the same FOA^R^/FOA^S^ equilibrium in 40 generations (Figure 2A). Hence, TPE assays based solely on the assessment of the *Y_S_*/*Y_A_* equilibrium give little information on the frequency of epigenetic conversions. Even more, the assessment of effects on gene silencing is credible only if evidence for dynamic equilibrium is produced, something that is rarely done.

We used the aforementioned considerations to empirically measure the frequency of *URA3* conversions at the *VIIL* telomere in wild-type (*W303*) cells. The cells were selected in parallel on both SC/FOA and SC-ura plates and then transferred to non-selective YPD medium. Aliquots were taken out at known generation numbers after the removal of selection and the percentage FOA^R^ cells were measured and plotted (Figure 2B). Best fit algorithm produced values of *C_S→A_* = 8.0 and *C_A→S_* = 6.3.

### Simulation of loss or gain of silencing

The reduction in the proportion of FOA^R^ cells to 1% or below is widely used as key evidence for loss of gene silencing at the telomere (2,37,38). However, it is not clear whether this shift of equilibrium is caused by increase of *C_S→A_* at fixed *C_A→S_* or if both parameters change. This uncertainty poses a technical challenge. If *C_A→S_* is reduced to 0.01% and *C_S→A_* increases to 15%, equilibrium will be reached in <30 generations. However, if *C_S→A_* remains at 8%, the equilibrium will be reached after 50 generations (Figure 2C). Similar dynamics can be projected for previously observed situations of gain of silencing, where FOA^R ^= 85% or higher (39–41) (Figure 2D).

### Simulation of loss or gain of epigenetic stability

In the preceding section, we have assumed that *C_A→S_* increases or remains constant, whereas *C_S→A_* decreases, or *vice versa*. However, our model allows for independent variations of these two parameters (Appendix 1). We therefore simulated situations where both parameters increase or decrease.

If *C_A→S_* and *C_S→A_* both increase to 15%, equilibrium will be reached within 10 generations (Figure 3A). This situation corresponds to epigenetic instability that exceeds any observed PEV phenomenon and would be difficult to detect by currently existing assays. In the case of *URA3*-based TPE analyses, this situation will be accompanied by slow growth of the cells on both SC-ura and SC/FOA media.

If *C_A→S_* and *C_S→A_* both decrease to 1%, the dynamic equilibrium will be reached after ≥100 generations (Figure 3B). Such a scenario defines a substantial gain in epigenetic stability. Assessment of the percentage of FOA^R^ will strongly depend on the initial (SC-ura or SC/FOA) selection used. Importantly, if selection has been performed only on SC-ura plates (as in many studies), the data will serve as evidence for loss of gene silencing, not for gain of epigenetic stability.

In summary, parallel experiments with selection for the active and silent state of the gene (SC/FOA and SC-ura in the case of *URA3*) are essential when significant differences between the A→S and S→A rates are expected or if these rates are unknown. In practice, a single time point measurement of *Y_A_* and *Y_S_* at the 20th generation can serve as evidence for equilibrium (or lack of equilibrium) and as an indicator, but not evidence for substantial changes in the frequency of epigenetic conversions.

### *CAC1* regulates the frequency of epigenetic conversions at the telomeres

**Figure 5.**
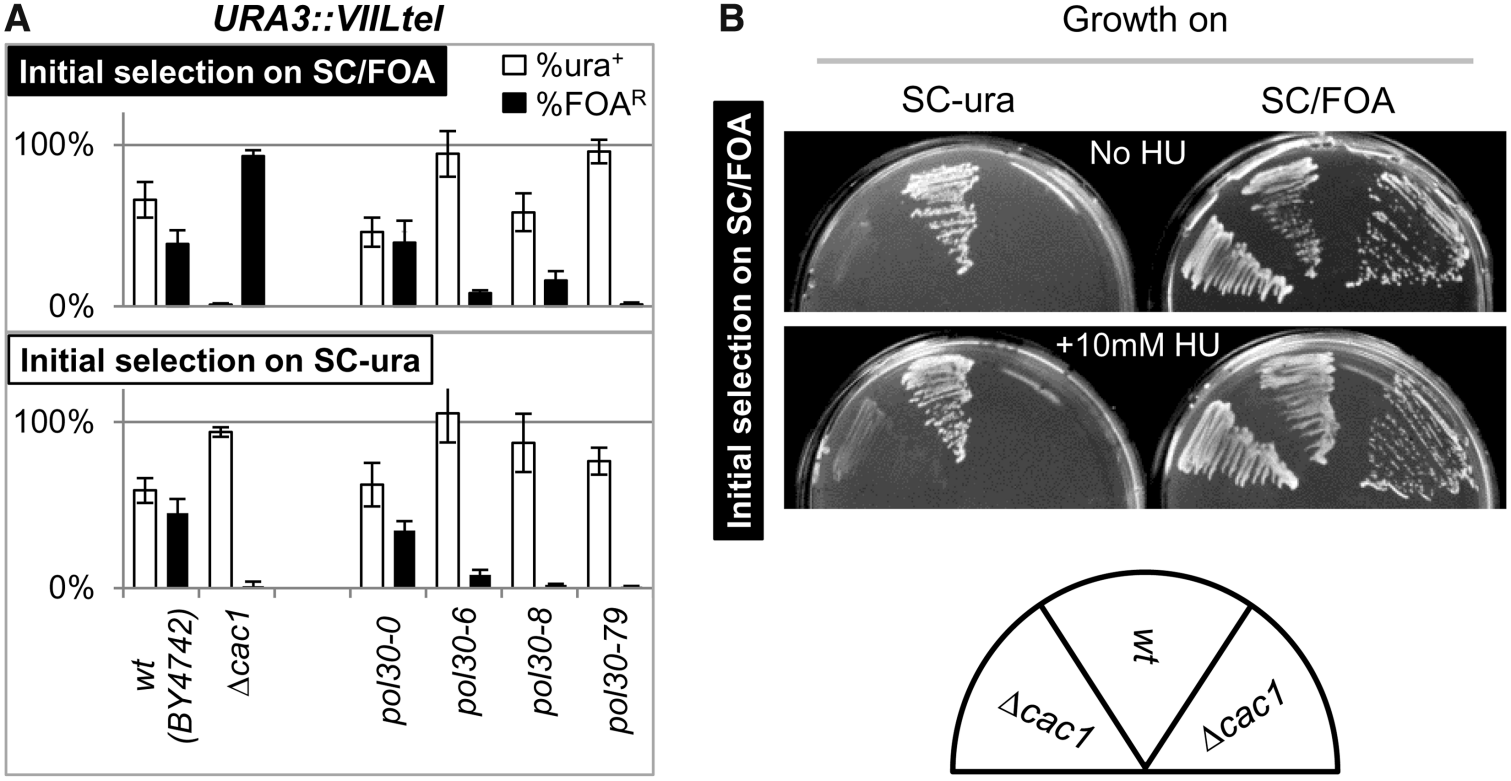
Effects of *POL30*(PCNA) and HU on epigenetic conversions. (**A**) Frequency of conversions at the *VIIL* telomere in *pol30(PCNA)* mutants. Analyses were performed as in Figure 4A. In the *pol30* mutants, the genomic copy of *POL30* is deleted, and viability is restored and maintained by *ARS/CEN/TRP1* plasmids containing wild-type (*pol30-0*) and mutant (*pol30-6*, *pol30-8*, *pol30-79*) alleles. Wild-type *(BY4742)* and *Δcac1* cells are shown for comparison. (**B**) Effect of HU on conversions at the *VIIL* telomere. *Δcac1* and isogenic wild-type *BY4742* cells containing *URA3* in the *VIIL* telomere were selected on SC/FOA plates and then transferred to SC-ura and SC/FOA plates containing 10 mM HU or to control SC-ura and SC/FOA plates as indicated. The *Δcac1* cells on the left-hand side of the wild-type cells have been grown for ∼10 generations in non-selective medium. The *Δcac1* cells on the right-hand side of the wild-type cells were re-streaked directly from SC/FOA plates.

We used the aforementioned considerations to search for genes that affect the frequency of epigenetic conversions. *URA3* was inserted in the *VIIL* telomere of several strains with previously reported deviations in gene silencing (Figure 4A) (36,37,39,42). The transformed cells were then selected on SC-ura and SC/FOA plates, transferred to liquid non-selective medium and grown for 20 generations. Aliquots of these cultures were then serially diluted and plated in parallel onto non-selective, SC-ura and SC/FOA plates. The proportions of URA^+^ and FOA^R^ cells (measured as the number of colonies that survived on selective SC-ura and SC/FOA media, respectively, divided by the number of colonies on the non-selective plates) were then calculated (Table 2) and plotted (Figures 4 and 5).

Relative to the wild-type counterpart, most of these strains showed an increase or decrease in the proportion of FOA^R^ cells, regardless of the initial selection on SC/FOA or SC-ura plates. For example, *RIF1* counteracts the association of Sir proteins with the telomere-bound Rap1 and has general anti-silencing activity (43,44). As expected, its deletion led to an increase in the %FOA^R^ cells (Figure 4A). On the other hand, mutations in *SAS2* and *SAS3* are known to reduce silencing at the telomere. As expected, their deletion reduced the %FOA^R^ values. The other mutants in Figure 4A have shown loss of silencing in many earlier studies, but the mechanisms of action for some of them are not completely understood (37,39). Importantly, the similar %FOA^R^ and %FOA^S^ values at the 20th generation after selection indicated that all these cultures were approaching or had already reached the point of dynamic equilibrium. Hence, all these strains displayed loss of silencing consistent with the simulations presented in Figure 2C and D, respectively. We did not attempt to precisely measure the frequency of conversions.

The *Δcac1* strain presented a notable exception. Its %FOA^R^ was strongly dependent on the initial selection (Figure 4A). This phenotype was consistent with the scenario depicted in Figure 3B and suggested lower rates of *URA3* conversions and gain of epigenetic stability at the *VIIL* telomere. We followed up by measuring the %FOA^R^ cells at multiple time points after the removal of selection. Best fit analyses of the data from the first 50 generations indicated that for this period, *C_S→A_* = 0.44% and *C_A→S_* = 0.06% (Figure 4B). Surprisingly, the continuing culturing of the cells in non-selective medium showed a sharp decline in the proportion of %FOA^R^ cells after the 60th generation. This decline was observed in cells selected on both SC/FOA and SC-ura (Figure 4B). By the 100th generation, FOA^R^ cells appear at <0.01%, regardless of the initial selection. In contrast, all other freshly transformed mutant strains (Figures 4A and 5A) showed low %FOA^R^ in <20 generations after removal of selection. Although we do not completely understand this rapid loss of telomeric repression in later *Δcac1* passages, we suspect that it could be related to the reduced level of Sir2p in aging cells as observed earlier in (45,46).

We also tested whether the observed suppression of epigenetic conversions in the early passages after selection applies to another sub-telomeric locus. We inserted *URA3* in the right arm of chromosome *V* as in (27) and conducted analyses as in Figure 4A. Again, *Δcac1* cells that were selected on SC-ura produced low %FOA^R^, whereas cells selected on SC/FOA showed the opposite trend (Figure 4C). In conclusion, we have acquired solid evidence for enhanced epigenetic stability at the *VIIL* and the *VR* telomeres in *Δcac1* cells.

*CAC1* encodes a component of the histone chaperone CAF-I and plays an important role in the replication-coupled assembly of nucleosomes (47). We considered the possibility that any general perturbation of histone exchange and/or nucleosome assembly could have an inhibitory effect on TPE. To address this question, we tested the effect on epigenetic stability at the *VIIL* telomere in other histone chaperone mutants. Experiments were performed exactly as in Figure 4A. We show that the deletion of *ASF1* and *HIR1* only slightly altered TPE (Figure 4D). Interestingly, the *Δrtt106* mutant had a minor gain of epigenetic stability, but not as strong as the *Δcac1* mutant. We also considered that the observed effects are indirect, and that in the absence of *CAC1*, some other chaperone plays a key role in reducing the frequency of epigenetic conversions. In Figure 4D, we show that double deletion of chaperone-encoding genes *Δcac1Δrtt106, Δcac1Δasf1* and *Δcac1Δhir1* produced phenotypes similar to that of *Δcac1*, whereas *Δhir1Δasf1* had little effect on epigenetic conversions. Hence, loss of variegation in *Δcac1* cells cannot be restored by deletion of other chaperones. These observations imply that *CAC1* directly contributes to PEV at the telomeres.

### *POL30*(proliferating cell nuclear antigen) and ribonucleotide reductase effects on epigenetic conversions

It is believed that CAF-I is recruited to replication forks via interactions with Pol30p(proliferating cell nuclear antigen) (47). *POL30* is involved in multiple transactions at the replication forks (48). In certain *POL30* mutants, a correlation between gene silencing and impaired binding to CAF-I has been observed (36,49). Specifically, the *pol30-6*, *pol30-8* and *pol30-79* mutants all display reduced telomeric silencing, whereas in biochemical assays, Pol30-8p and Pol30-79p poorly associate with Cac1p (36). We tested whether these *POL30* mutants display a phenotype similar to *Δcac1*. The experiments in Figure 5A revealed that *Δcac1* and the *pol30* mutants share the loss of silencing. However, only *Δcac1* suppressed variegation at the *VIIL* telomere. Although these results do not exclude the possibility that the interaction of CAF-I with Pol30p is important for TPE, they clearly show that only the loss of CAF-I contributes to variegation. Hence, there is no complete overlap between the TPE phenotypes of *pol30* and *cac1* mutants.

Recent studies have suggested that the toxicity of 5-FOA, while being primarily caused by the *URA3-*encoded Orotidine-5′-Phosphate-Decarboxylase, could be enhanced by the stimulation of ribonucleotide reductase (RNR) (14,50,51). Even more, suppression of RNR by low concentration of HU could reverse the sensitivity of *dot1*, *pol30* and *Δcac1* mutants to 5-FOA (51–53). We considered the possibility that RNR and HU could have a role in the frequency of epigenetic conversions in *Δcac1* cells and performed experiments similar to the ones in (51,52). First, we confirmed that under our experimental conditions, the sensitivity to 5-FOA strictly depends on the expression of *URA3* (Supplementary Materials, Figure 1). Next, we streaked *Δcac1* and isogenic *BY4742* cells on SC/FOA plates with and without 10 mM HU and grew them for 3 days at 23°C (Figure 4B). At this concentration, HU moderately reduces the dNTP pools, but does not induce S-phase arrest (51,52,54,55). Our data indicate that HU did not suppress or reverse the gain of epigenetic stability of *URA3* (Figure 5B). Similar data were obtained with cells that were initially selected on SC-ura plates (not shown). Therefore, it seems unlikely that RNR contributes to the reduced rate of epigenetic conversions in *Δcac1* cells.

We also tested whether 5-FOA and HU increase the mutation rates in *Δcac1* cells and whether these can alter the resistance to 5-FOA and mimic epigenetic stability. We used a routine assay for the accumulation of forward mutations in *CAN1* (56–58). *CAN1* encodes an arginine transporter and is not related to RNR or *URA3*. We found that the mutation rates in *Δcac1* cells do not exceed 1 × 10^−^^6^ (Supplementary Materials, Figure 2). Such rates can have only negligible contribution to the generation of 5-FOA-resistance of *Δcac1* cells with telomeric *URA3*, which occur at frequency of 10^−^^2^ to 10^−^^3^. Even more, these FOA^R^ cells revert to expression of *URA3* and to sensitivity to 5-FOA, albeit at significantly lower rates compared with wild-type cells (Figure 4B). We also found that 5-FOA, HU or the combination of both does not increase the yield of mutations (Supplementary Materials, Figure 2).

Earlier reports have shown significant, but not complete, de-repression of telomeric *URA3* in *Δcac1* mutants (36,49). A similar observation has been made in *Δsas2* and *Δsas3* cells (39,59,60). On the other hand, we have observed stable resistance to FOA in *Δcac1* cells after selection on SC/FOA. We tested whether loss of the *URA3* reporter could account for the resistance to FOA in *Δcac1*, *Δsas2* and *Δsas3* cells. We picked up colonies from YPD, SC-ura and SC/FOA plates and analyzed them by PCR with primers specific for *URA3* at the recombinant *VIIL* telomere. These assays showed that all cells continue to harbor *URA3* when grown on SC/FOA plates (Supplementary Materials, Figure 3). We conclude that our observations could not be attributed to the loss of the reporter.

### Different frequencies of conversion at *HMR*a

**Figure 6.**
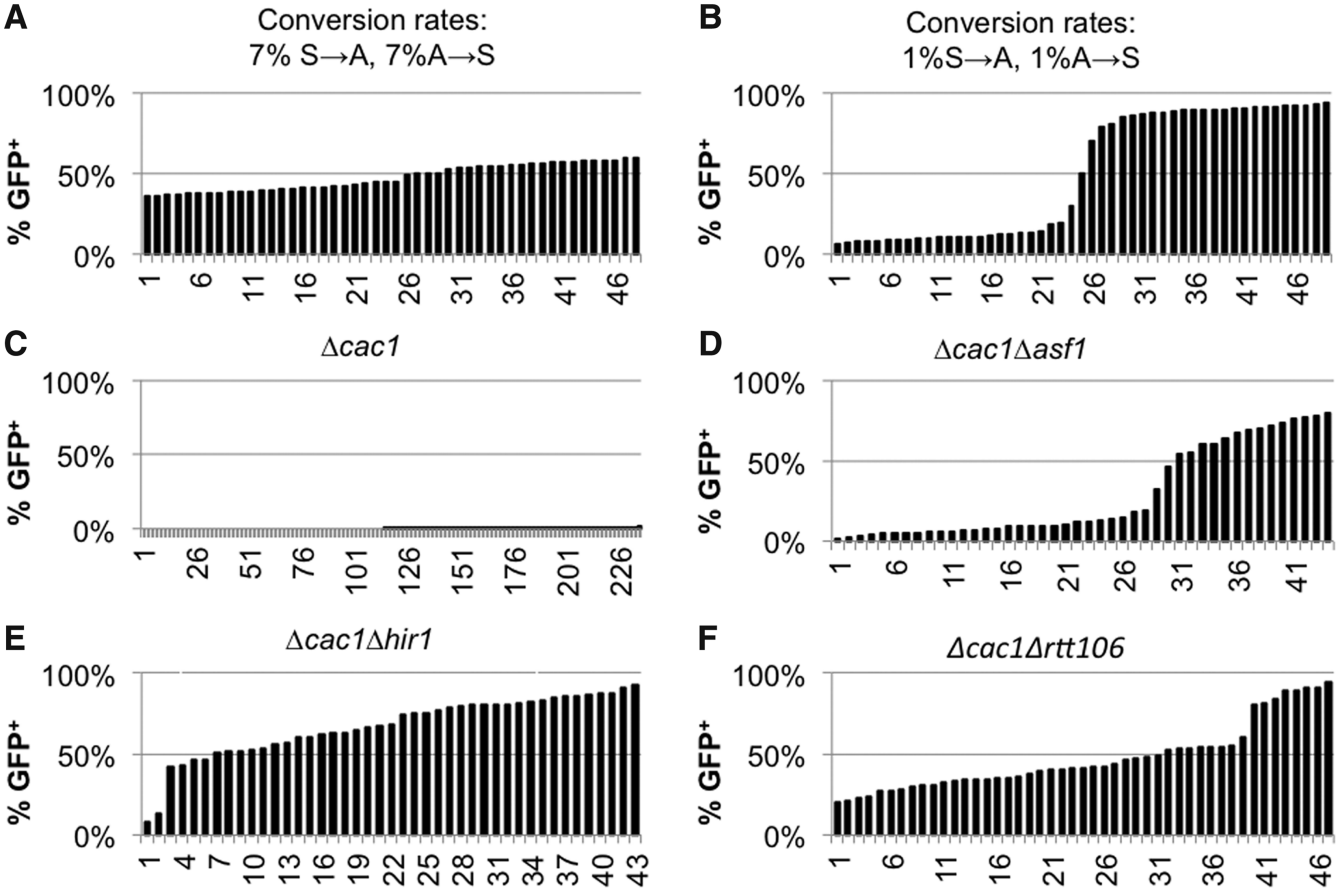
Assessment of the frequency of conversions at the *HMR***a***::URA3p::GFP* locus. Cells were seeded in 96-well plates at <1 cell per well and grown for 15–18 generations at 23°C. Wells with a single cluster of cells were identified and the proportion of GFP^+^ cells was assessed by FACS. The %GFP cells from each clone were individually entered in a spreadsheet, and the values were sorted and plotted as separate columns. About 45–50 individual clones were analyzed in (**D**–**F**) and 226 clones were analyzed in (**C**). (**A**) Simulation of the process at conversions rates of *C_S→A_* = 7% and *C_A→S_* = 7%. (**B**) Simulation of the process at conversions rates of *C_S→A_* = 1% and *C_A→S_* = 1%. (C–F) Experiments were performed with the strains listed on the top of the graphs.

We asked whether variations in the frequency of epigenetic conversions can be observed at another extensively studied locus, *HMR***a**. We made use of a modified *HMR***a** that contains a GFP reporter under the control of the *URA3* promoter (28). In wild-type cells, the genes in *HMR***a** (including this GFP construct) are permanently silenced. However, mutations in many genes lead to a variegated mode of expression (2,15,28). Such strains provide the means to measure gene silencing in the absence of 5-FOA and to alleviate the risk of its potential side effects (14,51), but no means for drug-based selection of the silenced state. For this reason, we diluted and dispensed such strains in 96-well plates to produce mini-cultures, which originate from a single cell, and then grew them for ∼15 generations. Depending on the state of the GFP reporter in the seeding cell, the initial value of the Y_(A)0_ is set at 100 or 0%. We simulated this process for 50 parallel mini-cultures (see Supplementary Materials, Appendix 2) and plotted the produced Y_(A)15_ values in Figure 6A and B. Because the cultures start from a single cell, the first S→A or A→S transition can occur anywhere in generation 1–6, thus producing significant variations in Y_(A)15_. With assumed rates of *C_S→A_* and *C_A→S_* of 7%, these values distribute linearly between 36 and 61% (Figure 6A), whereas reduction of the *C_S→A_* and *C_A→S_* conversions rates to 1% produces a distinct two-phase curve (Figure 6B).

Next, we performed such experiments in *Δcac1*, *Δcac1Δrtt106*, *Δcac1Δasf1* and *Δcac1Δhir1* cells harboring the *HMR***a***::URA3p::GFP* reporter (Figure 6C–E). Previous studies have shown that a single deletion of any of these chaperone genes, including *CAC1*, does not de-repress the *HMR***a***::URA3p::GFP* locus (28,30). However, in double deletion mutants, the locus acquires variegated mode of expression (28). In agreement, in the analyzed 226 *Δcac1 HMR***a***::URA3p::GFP* clones, we detected 176 clones with no GFP^+^ cells (Figure 6C and not shown). In the remaining 50 clones, the proportion of GFP^+^ cells varied between 0.2 and 0.9% (Figure 6C). This low incidence of GFP^+^ cells points to robust silencing with no conversions at all or to low S→A rate and precludes further analyses. In the double deletion mutants, we observed curves that suggest substantial differences in the conversion rates. Similar to the simulation in Figure 6B, the *Δcac1Δasf1* mutant displayed low conversion rates of ∼1% per generation. The *Δcac1Δhir1* and *Δcac1Δrtt106* mutants showed profiles of conversions rates that are in between the scenarios in Figure 6A and B. These results did not distinguish whether *CAC1* or other chaperones play a primary role in the execution of the conversions. However, they clearly demonstrated that the epigenetic transitions at this locus proceed at different rates in different strains. In addition, these experiments also indicated that different sets of genes could be involved in the control of epigenetic conversions at *HMR***a** and at telomeres.

### Rates of conversions in *Δsir1* strains

**Figure 7.**
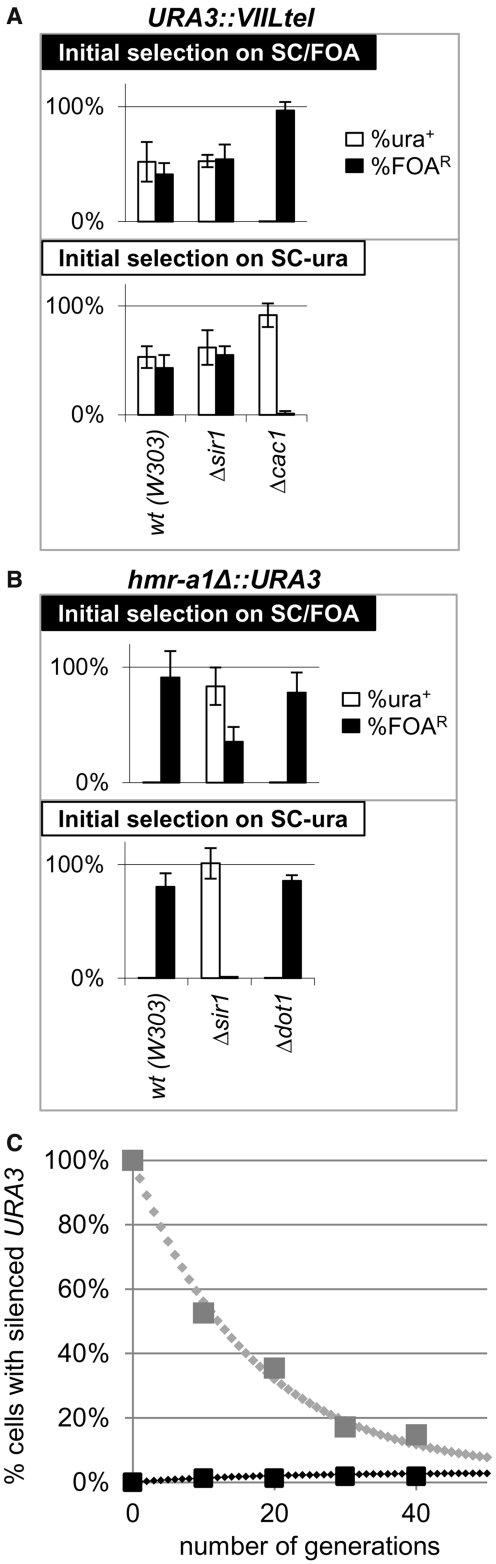
Frequency of conversions in *Δsir1* cells. *Δsir1*, *Δcac1* and wild-type cells harboring *URA3* at the *VIIL* telomere and *Δsir1*, *Δdot1* and wild-type cells harboring *hmr-a1Δ::URA3* were selected on SC-ura and SC/FOA plates, grown in YPD medium for 20 generations, and analyzed as in Figure 4A. (**A**) Analysis of cells with *URA3-tel.* (**B**) Analysis of cells with *hmr-a1Δ::URA3*. (**C**) Best fit analysis of the conversion rates in *Δsir1 hmr-a1Δ::URA3* cells. Cells were selected on SC/FOA (large gray squares) and SC-ura plates (large black squares) and transferred to YPD medium. Aliquots were taken out at known generation numbers and the percentage FOA^R^ cells were measured and plotted. Best fit algorithm (small diamonds) produced values of *C_S→A_* = 5.66% and *C_A→S_* = 0.17%.

Silent information regulator 1 (*SIR1*) is one of the few genes that have been implicated in the control of epigenetic conversions. Its deletion derepresses *HMLα* and *HMR***a** and produces higher S→A than A→S conversion rates at both of these loci. However, the precise calculated rates differ depending on the assays used (15,16,18). We tested the validity of our model using strains with *HMR***a** loci, which contain a *URA3* reporter and a destroyed Abf1p site in the *E* silencer *(hmr-a1Δ::URA3*) (16). Separately, we produced a *Δsir1* strain with an integrated *URA3* in the *VIIL* telomere exactly as in all other strains tested in Figure 4A. Because this *URA3-tel* construct does not contain *ARS* [*ARS* binds origin recognition complex (ORC) and is critical for the recruitment of Sir1p (2)], this strain is not expected to display *SIR1*-dependent effects on *URA3*. On the other hand, it has been demonstrated that *hmr-a1Δ::URA3* remains almost completely repressed in wild-type and *Δdot1* cells but is de-repressed in *Δsir1* cells (16). As predicted, experiments in cells with telomeric *URA3* showed no effect of *SIR1* and the characteristic low rates of conversion in *Δcac1* cells (Figure 7A). In *Δsir1 hmr-a1Δ::URA3* cells, the initial selection on SC-ura produced predominantly de-repressed *URA3*, whereas the cells selected on SC/FOA produced a mixed population of cells with active and silent *URA3* (Figure 7B). A time-course experiment with *Δsir1* cells revealed S→A rates of 5.7 and A→S rates of 0.17% (Figure 7C). This result is in good agreement with the higher S→A than A→S rates reported in (15,18) and with the overall reduced silencing of *hmr-a1Δ::URA3* reported in (16). Again in agreement with (16), *Δdot1* and wild-type cells generated only rare colonies on SC-ura plates, and these did not maintain the de-repressed state (Figure 7B). Hence, our experimental system has faithfully recaptured earlier observations and has confirmed the validity of our findings in *Δcac1* cells.

## DISCUSSION

In this study, we have addressed an important aspect of PEV: the frequency of conversions at PEV loci. In essence, position-effect phenotypes are determined by rare S→A and A→S conversions. In turn, the acquired states of the gene are epigenetically maintained through multiple cell divisions (5). In metazoans, such conversions could represent the point of tissue-specific epigenetic repression/activation of a gene. The acquired state could persist throughout the life of the organism. Untimely conversions (meaning to subject the locus to an S→A or A→S switch) lead to cancer and to a variety of genetic and psychiatric disorders (7–11). In contrast, stem cells maintain their functional plasticity via maintaining the ability to convert the epigenetic state of numerous genes (61,62). Given the significance of epigenetic conversions, it is surprising how little we know about them. In this study, we contribute to this specific field of genetics.

### Methodology

One of the important aspects of our study is methodological. The loose grip on the mechanisms of epigenetic conversions is in part due to the limited use of assays, which directly test the dynamics of these processes. For example, a few earlier reports have analyzed epigenetic switches at the mating type loci of *S. cerevisiae* (15–18). These have used specialized mating assays (‘shmoo’ farming and ‘alpha-factor confrontation’) or alternative assays with GFP and YFP reporters. Interestingly, the conversion rates measured by these approaches seem considerably different and may lead to different interpretations regarding the role of *SIR1*. Studies in another organism, *P. falciparum*, have extensively documented on- and off- switching rates of the subtelomeric *var* genes (23,25,26). However, their methodology accounts for the substantially more complex situation of multiple *var* loci and monoallelic exclusion and is not applicable to simpler cases (21,63,64).

In this report, we introduce a comprehensive model for PEV and project various scenarios based on independent rates of conversions at a single locus. This model represents an extension of the concepts outlined in (15,18,23). Our model (Figure 1) is applicable to any PEV locus and any organism. The projections based on this model provide solid definitions for the frequently used terms ‘loss’ and ‘gain’ of gene silencing (Figure 2). More importantly, we have also introduced the terms ‘loss’ and ‘gain’ of epigenetic stability (Figure 3) and have defined them graphically and through calculations of the coefficients of conversions *C_A→S_* and *C_S→A_*. Through simulations, we have shown how ‘loss of silencing’ and ‘gain of epigenetic stability’ can be confused if the assays do not specifically address the frequency of epigenetic switches. Finally, we introduce solid quantitative criteria and methodology for the precise measurement of the frequency of A→S and S→A conversions at any PEV locus (Figures 2 and 3). Our methodology can be used for the deciphering of the mechanisms of epigenetic switches in simple model systems and then be applied to more complex scenarios. An example of such applications is provided by revisiting the earlier experiments at the mating type loci of *S. cerevisiae* in *Δsir1* cells (15–18). ‘Shmoo’ farming and ‘α-factor confrontation’ had indicated that equilibrium of silencing at the *HMLα* locus was approached after ∼60 generations (15). A similar study with GFP and YFP had determined S→A rates of 13% and A→S rates of 8% when fluorescence was measured by FACS, or 35 and 15%, respectively, when fluorescence was measured for three generations by microscopy (18). According to our simulations, the first set of conversion rates will produce an S/A equilibrium in ∼20 generations (Figure 2A) and is consistent with loss of gene silencing. However, the set of short-term rates suggests that equilibrium will be reached in <10 generations (Figure 3B) and is consistent with loss of epigenetic stability. Yet, the long-term ‘α-factor confrontation’ assays clearly show extended epigenetic stability. Ultimately, these results indicate that short-term experiments depict a process that contributes to, but is not identical with, a long-term epigenetic conversion. Similar conclusions have been reached by (16,17,65).

Using our model, we have substantially modified two existing assays for gene silencing in *S. cerevisiae* to exclusively focus on the rate of conversions from repressed to active state and *vice versa*. These assays have shown that we can reliably detect ‘gain of epigenetic stability’ in this model organism, and that the histone chaperone CAF-I is involved in the regulation of epigenetic switches at the telomeres (Figure 4). Hence, our theory and methodology can be used for the identification of genes specifically involved in epigenetic conversions. The analysis of the mating type *HMR***a** locus does not provide the same level of confidence on the involvement of *CAC1*. Indeed, previous studies have indicated that histone chaperones can play different roles at the mating loci and at the telomeres (28,49). Still, the analyses in Figure 6 provide evidence for different conversion rates at a non-telomeric locus by a completely independent assay.

Many earlier studies have reported suppression of variegation because of contraction of a heterochromatin domain or because of repositioning of a hypothetical chromatin boundary (6,13). Such phenomena should not be linked to the switching mechanism. On the other hand, many studies have shown increased levels of expression of otherwise silenced reporters [reviewed in (3,12)]. These observations have often been attributed to ‘poor maintenance’ of gene silencing, meaning an elevated rate of S→A switches, or to incomplete repression of the gene in the PEV locus. As we have shown in Figure 3A, ‘poor maintenance’ is hard to detect, leaving these alternatives open to interpretation. A decrease in the rate of switching is a more reliable criterion for deregulation of positional variegation (Figure 3B). To date, only two articles have reported ‘enhanced memory for heritable transmission’ (19,20). These studies have shown that mutations in Histone H4 increase the stability of both the repressed and the transcribed states of telomeric reporters. Here, we show that the deletion of *CAC1* has similar effects (Figure 4). In this vein, the principle role of CAF-I is to reassemble Histones H4 and H3 after the transition of a replication fork (47).

### Role of CAF-I in epigenetic conversions

In theory, any perturbation of DNA replication and especially of fork integrity could lead to aberrant reassembly of chromatin (66). However, such events do not necessarily lead to enhanced or reduced epigenetic stability. For example, many of the strains in Figures 4A and 5A harbor mutations in core replication factors with well-established defects in DNA replication, but none of them showed the gain in epigenetic stability that we see in *Δcac1* mutants. The same reasoning applies to nucleosome assembly factors. The deletions of *rtt106*, *asf1* and *hir1* had little effect on TPE when compared with *Δcac1* (Figure 4D). Hence, the gain of epigenetic stability at telomeres is specific to *CAC1* and is not related to defects in DNA replication or nucleosome assembly in general.

Recent studies have raised the possibility that the loss of silencing in *pol30* and *cac1* mutants, which has been observed via telomeric insertion of *URA3* and the use of 5-FOA, could be produced by elevated RNR activity and unbalanced pools of dNTPs (51,53). Therefore, could the gain in epigenetic stability in *Δcac1* be linked to elevated RNR? Several arguments suggest the opposite. First, if RNR is important for the observed effects, it should induce sensitivity to 5-FOA, regardless of the prior selection of the cells. However, we see no sensitivity but robust resistance to 5-FOA in cells that have been initially selected on 5-FOA. This resistance could not be attributed to elevated RNR. Second, RNR activity can be reversed by low concentrations of HU (51,52,67). We saw no effect of HU on the frequency of epigenetic conversions in cells with wild-type *CAC1* (*BY4742*) or in *Δcac1* cells (Figure 5). Third, RNR is stimulated by a variety of mutations that cause replication stress (50,54,55) including most of the mutants we used in this study (Figure 4). None of these mutations caused reduction of telomeric variegation (Figure 4). We do not exclude the possibility that the absence of *CAC1* in combination with RNR stimulation has a strong impact on telomeric silencing (14). However, we favor the idea that CAF-I rather than RNR is the key player in epigenetic conversions.

Many reports have shown that the deletion of *CAC1* reduces gene silencing at telomeres (28,36,49,51,52,68,69). However, they have not assessed the stability of the repressed state of *URA3* after selection on SC/FOA plates. Our experiments agree with the findings in these articles but also add the analyses of the repressed state. Consequently, according to our criteria (Figures 2 and 3), we observe a gain of epigenetic stability rather than a loss of silencing. On the other hand, short-term (1–3 generations) experiments in *Δcac1* cells have revealed extremely high rates of telomeric S→A conversions (40%) (70). Such rates would preclude the detection of FOA resistance in *Δcac1* cells; yet, we and many others can readily establish FOA^R^ colonies. We suspect that, similarly to *HMLα* in *Δsir1* (16,18), short-term experiments in *Δcac1* cells capture an intermediate silencing state that is not the same as a stable epigenetic switch.

One interesting aspect of the epigenetic stability in *Δcac1* cells is the loss of telomeric silencing after the 60th generation (Figure 4B). At this point, we do not entirely understand the basis of this phenomenon. Earlier reports have shown a reduction in the abundance of Sir2p and concomitant decline in telomeric gene repression in aging cells (45,46). We have not conducted experiments that can explicitly link *CAC1* to the replicative lifespan of the cells. This possibility remains open for future studies.

### Mechanism of epigenetic stability in *Δcac1* cells

At present, we could not address the mechanism of gain in epigenetic stability in *Δcac1* cells. CAF-I is thought to play a central role in the re-assembly of nucleosomes behind the replication forks and in the transmission of epigenetic states (47,71). Some preliminary data (R. Oshidari, D.J., B.W.) point to the possible involvement of pausing of replication forks in the conversion of epigenetic state. Interestingly, it is also known that replication forks tend to pause in the subtelomeric regions (72,73). It is tempting to speculate that when replication forks pause, CAF-I acts as a ‘lax’ chaperone and allows lenient assembly of nucleosomes. This situation provokes poor transmission of the pre-existing epigenetic marks and predisposes the locus to epigenetic conversions. The absence of CAF-I at such paused forks can explain the reduced rate of epigenetic conversions at the telomeres. More focused mechanistic studies need to address these issues.

## SUPPLEMENTARY DATA

Supplementary Data are available at NAR Online.

## FUNDING

National Science and Engineering Research Council (NSERC) of Canada [#217548-2010 to K.Y.]; a joint grant from Japan Society for the Promotion of Science (JSPS)/Canadian Institutes for Health Research (CIHR) [JOH 410-90177-08 to K.Y. and H.M.]. Funding for open access charge: NSERC of Canada [#217548-2010].

*Conflict of interest statement*. None declared.

## Supplementary Material

Supplementary Data
