# Supplementary material for: Analysis of epigenetic stability and conversions in Saccharomyces cerevisiae reveals a novel role of CAF-I in position-effect variegation
Source: Nucleic Acids Res. 2013 Jul 17;41(18):8475–88. doi: 10.1093/nar/gkt623 (PMC3794585; doi:10.1093/nar/gkt623)
Supplement: Supplementary Data [file supp_41_18_8475__index.html]

Analysis of epigenetic stability and conversions in Saccharomyces cerevisiae reveals a novel role of CAF-I in position-effect variegation — Analysis of epigenetic stability and conversions in Saccharomyces cerevisiae reveals a novel role of CAF-I in position-effect variegation — Supplementary Data 

# Analysis of epigenetic stability and conversions in *Saccharomyces cerevisiae* reveals a novel role of CAF-I in position-effect variegation

## 

files

**Files in this Data Supplement:**

- Supplementary Data - pdf file
