# Supplementary material for: Analysis of epigenetic stability and conversions in Saccharomyces cerevisiae reveals a novel role of CAF-I in position-effect variegation
Source: Nucleic Acids Res. 2013 Jul 17;41(18):8475–88. doi: 10.1093/nar/gkt623 (PMC3794585; doi:10.1093/nar/gkt623)
Supplement: Supplementary Data [file supp_gkt623_nar-00419-x-2013-File009.pdf]

Figure 1. Toxicity of 5-FOA depends on the expression of *URA3* and is not reversed by Hydroxyurea.

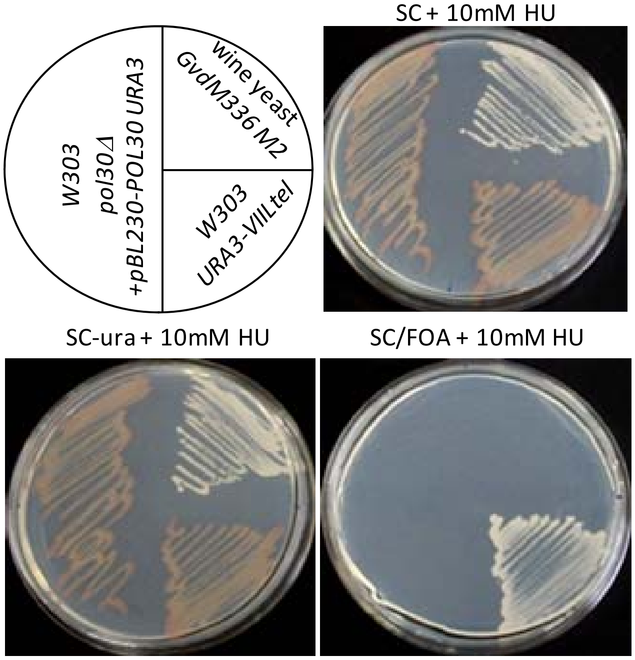

It has been shown that Hydroxyurea (HU) has different effects on DNA replication at different temperatures (1) and that HU can modulate the sensitivity to 5-FOA (2,3). It is possible that under certain conditions (different temperatures, different concentrations of Hydroxyurea and 5-FOA) *URA3*-expressing cells can survive in the presence of 5-FOA. We tested if under our experimental conditions toxicity to 5-FOA depends on the expression of *URA3*.

Wild type wine yeast (*URA3*), *W303 (ura3-52 URA3::VIILtel)* and *W303Δpol30* cells carrying *POL30* on a plasmid (*pBL230-POL30 URA3)* were streaked on SC, SC-ura and SC/FOA plates supplemented with 10 mM Hydroxyurea and grown at 23°C for 2 days on SC and SC-ura plates and for 6 days on SC/FOA plates. The variegating *URA3* at the *VIIL* telomere of *W303* cells confers growth on medium without uracil and on medium containing 2 μg/ml 5-FOA. In contrast, the plasmid-borne *URA3* confers growth on SC-ura. However, its loss renders the cells non-viable because of the concomitant loss of *POL30*. Wild type wine yeast cells are also non-viable indicating complete sensitivity to 5-FOA. The addition of 10 mM Hydroxyurea does not reverse the toxicity of 5-FOA after 6 days of incubation. We conclude that at the conditions used the toxicity of 5-FOA strictly depends on the expression of *URA3* and not on the activity of RNR.

Figure 2. Spontaneous mutation rates in *BY4742* and *Δcac1* cells in the presence of 5-FOA and HU. *CAN1* encodes for an Arginine transporter. Can1p also transports the toxin canavanine and confers sensitivity to the drug in the absence of arginine. Gain of canavanine resistance is frequently used as a measure for spontaneous mutations in *S. cerevisiae.* In wild type cells the yields of forward *CAN1* mutations is in the range of $3x10^{-7}$ (4,5). A genome-wide screen has identified non-essential genes whose loss moderately ($3x10^{-6}$) or severely (up to $1.67x10^{-5}$) increase these mutation rates (5). In *Δcac1* and *pol30* mutants these rates are in the range of 1-$3x10^{-6}$ (4,6,7).

We tested if 5-FOA or HU increase mutation rates in *Δcac1* cells and if the mutation rates change depending on whether the cells contain telomeric *URA3*. Liquid cultures of *Δcac1* or isogenic *BY4742* cells with or without *URA3* at the *VIIL* telomere, respectively, were grown in YPD and washed once in sterile water. 2-3 $x10^{7}$ cells were spread on SC-arg plates containing 60 μg/ml canavanine, 2 μg/ml 5-FOA or 10 mM HU as indicated. Plates were grown for 4 days and photographed. As demonstrated earlier (4,7), there is a moderate increase in the forward *CAN1* mutations in *Δcac1* cells (see the SC+Can plates). Such spontaneous mutation rates (less than 10^{-6}) could not account for the incidence of FOA-resistant cells (see SC/FOA plates) in *Δcac1* with telomeric *URA3* (10^{-2} to10^{-3}). We also show that 5-FOA, HU or the combination of both did not increase the mutation yields in cells without *URA3* and that the mutation rates in the presence of the drugs were not enhanced by the insertion of *URA3* in the telomeres. Hence, 5-FOA derivatives that are produced by the *URA3*-encoded Orotidine-5'-phosphate-decarboxylase do not increase mutation rates. We do not exclude that at significantly lower concentration of 5-FOA such derivatives could show mutagenic activity. However, in TPE assays the toxicity of 5-FOA seems to significantly exceed its potential mutagenicity.

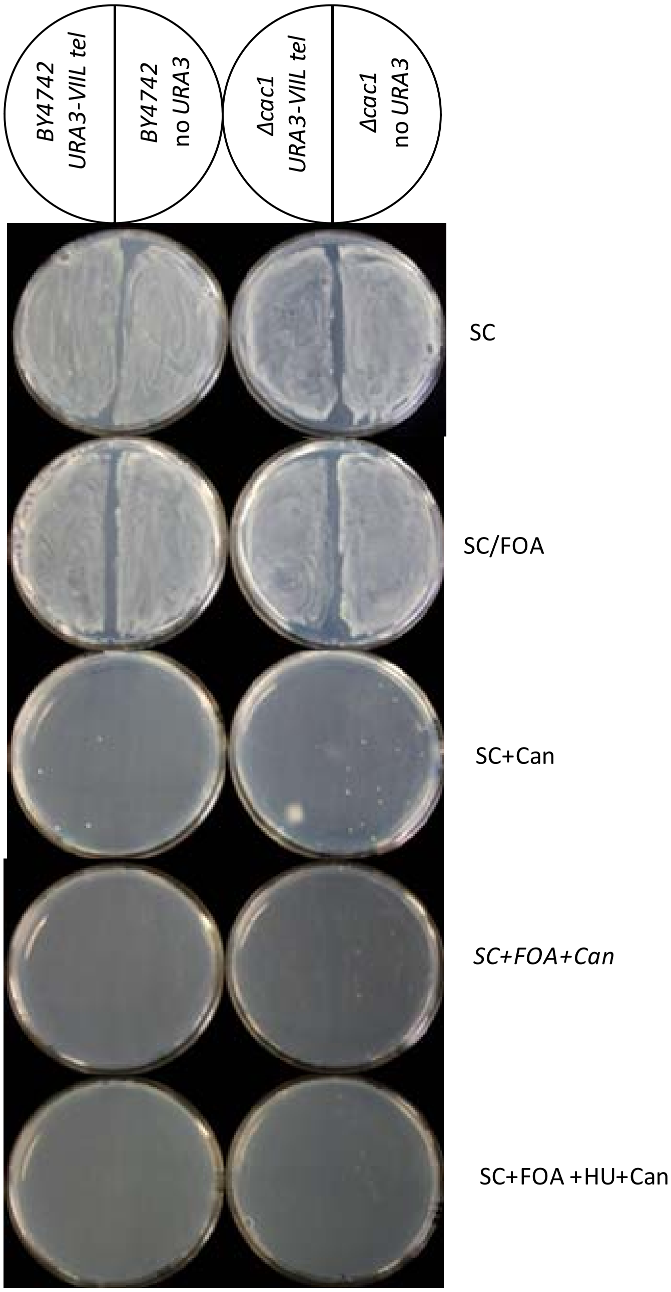

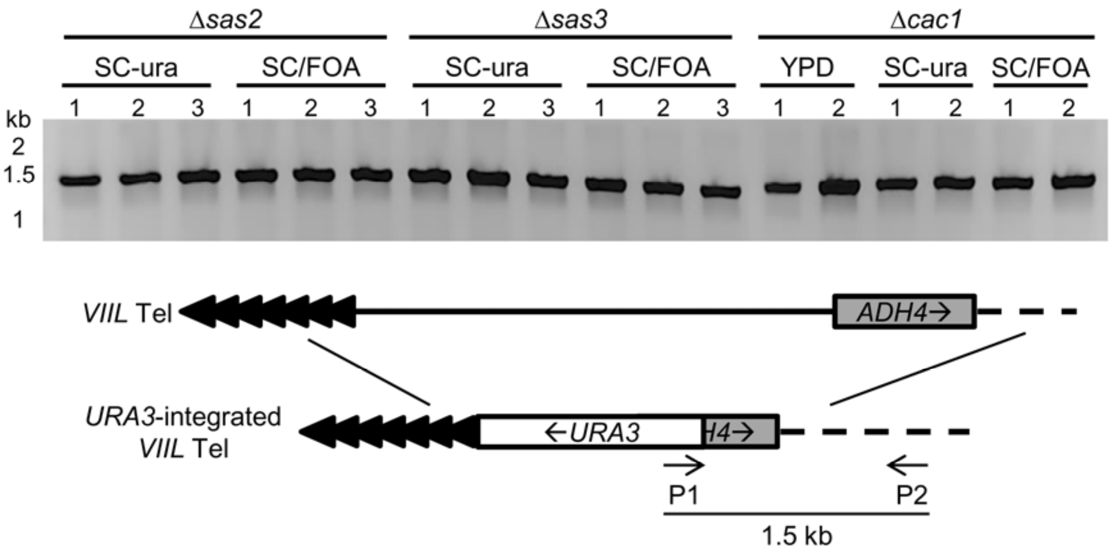

Figure 3. PCR confirmation for the presence of *URA3* at the *VIIL* telomeres in *Δsas2, Δsas3*, and *Δcac1 ADH4-URA3-tel* transformed cells after selection on SC-ura and SC/FOA. *Δsas2, Δsas3,* and *Δcac1* were transformed with the *ADH4-URA3-tel* construct and selected on SC-ura. A colony from each strain was grown in 3ml YPD and the cells spread on SC-ura, SC/FOA and YPD. Genomic DNA was isolated from individual colonies and PCR was performed with the primers as shown in the diagram. The sequence of the primers is available upon request. All colonies displayed the expected 1.5 kb PCR fragment regardless of selection on SC-ura or SC/FOA. We conclude that *URA3* was not lost upon selection on SC/FOA.

REFERENCES IN SUPPLEMENTARY FIGURES

1. Poli, J., Tsaponina, O., Crabbe, L., Keszthelyi, A., Pantesco, V., Chabes, A., Lengronne, A. and Pasero, P. (2012) dNTP pools determine fork progression and origin usage under replication stress. *EMBO J*, **31**, 883-894.
2. Poon, B.P. and Mekhail, K. (2012) Effects of Perinuclear Chromosome Tethers in the Telomeric URA3/5FOA System Reflect Changes to Gene Silencing and not Nucleotide Metabolism. *Front Genet*, **3**, 144.
3. Rossmann, M.P., Luo, W., Tsaponina, O., Chabes, A. and Stillman, B. (2011) A common telomeric gene silencing assay is affected by nucleotide metabolism. *Mol Cell*, **42**, 127-136.
4. Huang, M.E., Rio, A.G., Nicolas, A. and Kolodner, R.D. (2003) A genomewide screen in Saccharomyces cerevisiae for genes that suppress the accumulation of mutations. *Proc Natl Acad Sci U S A*, **100**, 11529-11534.
5. Davidson, M.B., Katou, Y., Keszthelyi, A., Sing, T.L., Xia, T., Ou, J., Vaisica, J.A., Thevakumaran, N., Marjavaara, L., Myers, C.L. *et al.* (2012) Endogenous DNA replication stress results in expansion of dNTP pools and a mutator phenotype. *EMBO J*, **31**, 895-907.
6. Myung, K. and Kolodner, R.D. (2003) Induction of genome instability by DNA damage in Saccharomyces cerevisiae. *DNA Repair (Amst)*, **2**, 243-258.
7. Myung, K., Pennaneach, V., Kats, E.S. and Kolodner, R.D. (2003) Saccharomyces cerevisiae chromatin-assembly factors that act during DNA replication function in the maintenance of genome stability. *Proc Natl Acad Sci U S A*, **100**, 6640-6645.

Appendix 1. Calculations on a recurrence relation of the type

$$\mathbf{Y_{(A)n} = Y_{(A)n-1} - Y_{(A)n-1}\, C_{(A\to S)} + (1- Y_{(A)n-1})\, C_{(S\to A)}}$$

where $Y_{(A)n}$ is the proportion of cells with **A**ctive gene at generation *n* (n_0… n_{-1}, n, n_{+1}... etc), $C_{(A\to S)}$ is the coefficient of conversions from **A**ctive to **S**ilent state and $C_{(S\to A)}$ is the coefficient of conversions from **S**ilent to **A**ctive, or simply presented as

$$Y_n = Y_{n-1}(1 - C_{(A\to S)}) + (1- Y_{n-1})\, C_{(S\to A)} \quad \text{or}$$

$$Y_n = Y_{n-1}(1 - C_{(A\to S)} - C_{(S\to A)}) + C_{(S\to A)}$$

I. The basic problem is to solve a first order recurrence relation,

$$X_n = aX_{n-1} + b \text{ for } n \geq 1 \qquad (1)$$

Where X_0, a and b are parameters that we specify in the model.

$$a = 1 - C_{(A\to S)} - C_{(S\to A)}$$

$$b = C_{(S\to A)}$$

In the initial condition (growth on selection medium) X_0 could be $X_0 = 1$ or $X_0 = 0$.

A second order recurrence relation would be $\boldsymbol{X_n=aX_{n-1} + bX_{n-2} + c}$. We will not be concerned with second order recurrence relations other than to say that the method we describe will apply to these and higher order recursions as well. We then apply the method of telescoping with the idea to write down the system of equations that the $\boldsymbol{\{X_n\}}$'s satisfy, and then to multiply each equation by $\boldsymbol{\{1, r, r^2,...)}$ in succession. The value of r is then chosen so that the variables $\boldsymbol{\{X_n\}}$ cancel from the system. As a first step, we write down the system of equations for the $\boldsymbol{\{X_n\}}$:

$$\begin{aligned} X_n &= aX_{n-1} + b \\ X_{n-1} &= aX_{n-2} + b \\ &\vdots \qquad\qquad (2) \\ X_2 &= aX_1 + b \\ X_1 &= aX_0 + b \end{aligned}$$

II. We keep the first equation, we multiply the second equation through by $\boldsymbol{r}$, the third equation through by $\boldsymbol{r^2}$… and so on, so that the last equation is multiplied through by $\boldsymbol{r^{n-1}}$. The system of equations now looks this way:

$$\begin{aligned} r^{n-1}X_n &= r^{n-1}(aX_{n-1} + b) \\ r^{n-2}X_{n-1} &= r^{n-2}(aX_{n-2} + b) \\ &\vdots \qquad\qquad (3) \\ r^2X_3 &= r^2(aX_2 + b) \\ rX_2 &= r(aX_1 + b) \\ X_1 &= aX_0 + b \end{aligned}$$

X_1 appears once on the left hand side of the system, and once, as raX_1, on the right hand side of the system. If we want X_1 to cancel from the system, we must choose r such that $1 = ra \Rightarrow r = \frac{1}{a}$.

X_2 appears once on the left hand side of the system as rX_2, and once on the RHS of the system as r^2aX_2. If we also want X_2 to cancel from the system, we must choose r such that $r = r^2a$. We may cancel r from both sides of this equation, and we obtain $1 = ra \Rightarrow r = \frac{1}{a}$. This pattern repeats

itself. Thus, the value of $r = \frac{1}{a}$ will cause all the variables $\{X_{n-1}, X_{n-2}, \ldots, X_1\}$ to cancel from the system. We put $r = \frac{1}{a}$, and add all the above equations in (3), and we are left with:

$$r^{n-1}X_n = aX_0 + b(r^{n-1} + r^{n-2} + \cdots + r + 1) \qquad (4)$$

where all the intermediate variables have cancelled from the system (this is called telescoping). Now $1 + r + \cdots + r^{n-2} + r^{n-1}$ is called a geometric series, and the formula for summing a geometric series gives us

$$1 + r + \cdots + r^{n-2} + r^{n-1} = \frac{1 - r^n}{1 - r} = \frac{1 - \left(\frac{1}{a}\right)^n}{1 - \left(\frac{1}{a}\right)} = \frac{a^n - 1}{a^n - a^{n-1}}$$

The previous equation follows from putting $r = \frac{1}{a}$, and then multiplying both numerator and denominator through by a^n. Using this equation to sum the geometric series in (4), and then multiplying through both sides of (4) by a^{n-1}, we obtain the solution for X_n:

$$X_n = a^n X_0 + b\left(\frac{a^n - 1}{a - 1}\right) \qquad (5)$$

III. With (5) in hand, we return to our initial recursion:

$$Y_n = Y_{n-1} - Y_{n-1}C_{(A\to S)} + (1 - Y_{n-1})C_{(S\to A)} = (1 - C_{(A\to S)} - C_{(S\to A)})Y_{n-1} + C_{(S\to A)}$$

It is clear that we should choose $a = 1 - C_{(A\to S)} - C_{(S\to A)}$ and $b = C_{(S\to A)}$. Putting these values in (5), we obtain

$$X_n = \left(1 - C_{(A\to S)} - C_{(S\to A)}\right)^n + C_{(S\to A)}\left(\frac{(1 - C_{(A\to S)} - C_{(S\to A)})^n - 1}{(1 - C_{(A\to S)} - C_{(S\to A)}) - 1}\right)$$

$$X_n = (1 - C_{(A\to S)} - C_{(S\to A)})^n + C_{(S\to A)}\left(\frac{1 - (1 - C_{(A\to S)} - C_{(S\to A)})^n}{C_{(A\to S)} + C_{(S\to A)}}\right) \qquad (6)$$

The last item to consider is what happens for large values of n, i.e., what is the convergence behavior as $n \to \infty$. A limit will only exist if

$$-1 < 1 - C_{(A\to S)} - C_{(S\to A)} < 1$$

which simplifies to give

$$0 < C_{(A\to S)} + C_{(S\to A)} < 2.$$

As long as we have this condition, we may take $\lim_{n\to\infty}$ in (6), and we obtain:

$$\lim_{n\to\infty} X_n = 0 + \frac{C_{(S\to A)}}{C_{(A\to S)} + C_{(S\to A)}}(1 - 0) = \frac{C_{(S\to A)}}{C_{(A\to S)} + C_{(S\to A)}} \qquad (7)$$

a ratio of the parameters.

Appendix 2: Assessment of variegation at the *HMRa::URA3p::GFP* locus

The assays described below are designed to assess the conversion rates of the *URA3p::GFP* with no prior selection. For this reason, we produce multiple cultures that originate from a single cell and then measure the proportions of GFP^+ cells in each of them. By definition, at seeding (this is generation "0", n_0) each culture must be either 100% GFP^+ or 0% GFP^+. In the first 6 generations these cultures will have 1 to 32 cells, respectively. As per Appendix 1, at conversion rates of 7% the S→A or A→S conversions are less likely to take place in the earlier rather than the later generations. However, any conversion in the earlier generations would have a significantly greater impact on the proportion of GFP^+ cells (this is the $Y_{(A)}$ value) than in subsequent generations.

The effects of the first conversion, depending on the generation, were calculated to partially account for this difference using the formula:

$$Y_{(A)n} = |Y_{(A)n-1} - 2/N_n|$$

where n is generation number, N is the number of cells, and $Y_{(A)}$ is the proportion of GFP^+ cells.

The likelihood (P_n) of the first A→S or S→A conversion occurring in each of the generations was calculated using the following formula:

$$P_n = N_{n-1}C\ (1- (P_{n-1} + P_{n-2} + P_{n-3} \ldots.)$$

where C is $C_{(A\rightarrow S)}$ if $Y_{(A)n-1} = 1$ or $C_{(S\rightarrow A)}$ if $Y_{(A)n-1} = 0$.

For example, at generation n=2, we will have only 4 cells. Since it is impossible to have 7% GFP^+ when each cell must be either 100% GFP^+ or 0% GFP^+, if the $Y_{(A)1} = 1$, then we would calculate $Y_{(A)2}$ as $|1 - 2/4| = 0.5$. Then, assuming a $C_{(A\rightarrow S)}$ of 7% for our model, we would calculate the likelihood of the first conversion occurring at generation n=2 as:

$$P_2 = 2*0.07\ (1- P_1)$$
$$= 2*0.07\ (1 - (1*0.07)$$
$$= 0.1302$$

These cell number effects were considered negligible after generation n=6 and all other values, as well as those following the "first conversions", were calculated using the formula from Appendix 1:

$$\mathbf{Y_{(A)n} = Y_{(A)n-1} - Y_{(A)n-1}\, C_{(A\rightarrow S)} + (1- Y_{(A)n-1})\, C_{(S\rightarrow A)}}$$

Using a simulation for a total of 50 mini-cultures, the P_n was used to calculate how many samples needed to be included from each "first conversion generation". Next, each sample $Y_{(A)}$ value was calculated for each generation up to the 18th generation in Microsoft Excel. Two scenarios were considered: silenced to active (S→A) and active to silenced (A→S) conversion rates of 7% each (similar conversion rates to the *VIIL* telomere in wild type cells) or of 1% each (similar conversion rates at the *VIIL* telomere in *Δcac1* cells). Finally, the $Y_{(A)}$ values starting from generation 15 were sorted in ascending order and used to build the distribution graphs in Figures 6A and 6B. The Excel sheets are available upon request.
